# Mycobacterium avium Complex Extracellular Vesicles Attenuate Inflammation via Inducing IL-10

**DOI:** 10.22088/IJMCM.BUMS.7.4.241

**Published:** 2019-04-07

**Authors:** Mohammad Kamran-Sarkandi, Ava Behrouzi, Aboulfazl Fateh, Farzam Vaziri, Mehdi Mirsaeidi, Seyed Davar Siadat

**Affiliations:** 1 *Department of Microbiology, Science and Research Branch, Islamic Azad University, Tehran, Iran.*; 2 *Department of Mycobacteriology and Pulmonary Research, Pasteur Institute of Iran, Tehran, Iran.*; 3 *Microbiology Research Center, Pasteur Institute of Iran, Tehran, Iran.*; 4 *Division of Pulmonary, Critical Care, Sleep and Allergy, Department of Medicine, University of Miami, Miller School of Medicine, Miami, USA.*

**Keywords:** Mycobacterium avium complex, extracellular vesicle, interferon gamma (IFN-γ), interleukin 10 (IL-10)

## Abstract

Mycobacterium avium complex (MAC) is an ubiquitous acid-fast bacterium. MAC cell wall and membrane release extracellular vesicles (EVs) into different media. The immunogenic effects of EVs isolated from MAC remain unknown. The aim of this study was to determine the EVs effect on macrophage cytokine production. MAC EVs were extracted and purified using differential centrifuges also known as Claassen’s method, with some modifications. After protein analysis of EVs, and scanning electron microscopy (SEM), the EVs were injected into BALB/c mice for in vivo experiments. The concentration of interferon gamma (IFN-γ) and interleukin 10 (IL-10) in the spleen immune cell culture was measured by sandwich ELISA. We for the first time showed that MAC can naturally produce EVs. The extraction method was technically-feasible, efficient and affordable. The SEM analysis showed that EVs diameter was similar to other studies on mycobacteria, and EVs maintained their spatial characterization. The results of the cytokine assays indicated that EV-treated cells secreted IL-10 (P = 0.034) but not IFN-γ (P = 0.037). Our findings suggest that EVs of M. avium could have anti-inflammatory effects. They can be used as a suppressor or regulator of inflammation via IL-10. The replication of the anti-inflammatory response of MAC EVs in future studies may indicate a new therapeutic agent for inflammation.

Mycobacteria are Gram-positive bacteria that cause significant global health problems. They are non-motile aerobic actinomycetes, rod-shaped, have lipid-rich cell envelope and their genome is rich in guanine- cytosine (69%) (1). Non-tuberculosis mycobacteria (NTM), especially *Mycobacterium avium* complex (MAC), infect healthy people (2) and patients with AIDS and other immunodeficiency diseases. MAC has more than 11 subspecies including* M. avium* and* M. intracellulare* (3). This bacteria is ubiquitous and can be isolated from water, soil, food, and animals especially birds (4). MAC rarely causes disease in people with a normal immune system (2). Mycobacteria envelope comprises various soluble proteins, carbohydrates, lipids, and insoluble macromolecules such as arabinogalactan, peptide-glycan, and mycolic acids (5). These components constitute a mycoyl-arabinogalactan-peptidoglycan complex which is one of the two common lipopolysaccharides (LPS) in mycobacteria (6). In addition, all mycobacteria have lipoarabinomannan as a part of their cellular envelope. Lipoarabinom-annan does not have a covalent bond with the mycoyl- arabinogalactan- peptidoglycan complex, but it may connect to the mycobacterial plasma membrane (6). Mycoyl- arabinogalactan-peptidoglycan complex, lipoarabinomannan, and C-mycosideglycopeptidolipids are more immunogenic than LPS of other bacteria (5). The cell envelope lipids and glycolipids are potent modulators of host macrophage cells which can induce pro-inflammatory chemokines and cytokines (7, 8). MAC infects a variety of cells and is linked to persistent infection in the immunocompromised hosts (9). Macrophages do phagocytosis of MAC via complement receptor 3 (CR3) (10). MAC is also attached to serum fibronectin and enters macrophages through the fibronectin-integrin receptor (11, 12). The activation of macrophages and inhibition of MAC growth depends on its strain; for instance, interferon-gamma (IFN-γ) facilitates the inhibition of certain non-AIDS strains of MAC, but not some isolates from patients with AIDS (13, 14). The immune system enhances IFN-γ when the host is confronted with mycobacteria, and it plays a strong role to prevent MAC growth, but IL-10 release facilitates MAC’s escape from Th1 response (15, 16).

Pathogenic bacteria use a different secretory system to release products into the cellular environment, tissue or the bloodstream of host organisms (17). Some bacteria, fungi, and archaea use membrane vesicles to release complex groups of proteins, polysaccharides, and lipids in the extracellular space (17-21). Extracellular vesicles (EVs) have a spherical structure that is surrounded by two layers of lipid similar to the cell membrane, and they contain hydrophilic components (22). These EVs were isolated for the first time from *S. aureus* and later it was shown that Gram-positive bacterial EVs share many common features with outer membrane vesicles (OMVs) of Gram-negative bacteria (23). EVs of mycobacteria were first isolated from the extracellular matrix of *Mycobacterium ulcerans* biofilms (24). The range of vesicle diameters was from 60 to 300 nm which is similar to that of OMVs produced by Gram-negative bacteria. EVs are composed of mycobacterial lipid and lipoprotein that originate from the plasma membrane (18).

EVs may carry a variety of components including virulence factors, DNA, RNA, toxins, immune modulatory factors, and food trapping factors like OMVs (17, 25). EVs stimulate cytokine production from macrophages and other immune cells, and consequently may induce immunity (26). The OMVs-based vaccine is getting more attention after *Neisseria meningitides* vaccine received a license from food and drug administration (FDA) as the first one in this category (27).

The interaction of MAC extracellular vesicles with the immune system is not well known. In this study, we aimed to isolate MAC EVs, and determine their effect on the immune system in a mouse model to understand in some context, the immunogenic response that is triggered in response to MAC.

In this study, we selected IFN-γ as a T-helper1 (Th1) response, and IL-10 as a T-helper2 (Th2) response, and illustrated which response is the dominant response when the EVs are injected to mice.

## Materials and Methods

**Media and bacterial cultures**

**Fig. 1:**
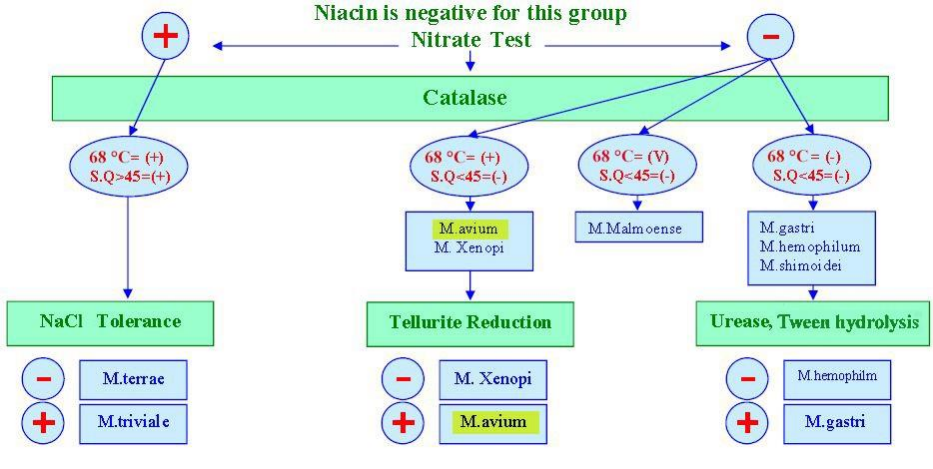
**Biochemical tests.** Niacin and nitrate tests were negative for *M. avium *complex. 22 and 68 °C catalase test were positive, but semi-quantitative (SQ) test was negative. Also, tellurite reduction test was positive

MAC (strain NO. CRBIP 7.142) was obtained from the Mycobacteriology and Pulmonary Research Department of Pasteur Institute of Iran, cultured in Lowenstein-Jensen (LJ) and Middlebrook 7H9 broth base (HiMedia, India) supplemented with 1 g dextrose and 0.0015 g catalase for 15 days at 37^ o^C. Identification of MAC was performed with phenotypic characteristics including growth rate, optimum temperature, pigment production, acid-fast staining, as well as biochemical tests. The biochemical tests included niacin, nitrate reduction, catalase (22 ^o^C, 68^ o^C), and semi-quantitative (SQ) test, tellurite reduction, urease, and NaCl tolerance. For microscopic detection, Ziehl-Neelsen staining was applied. A summary of the biochemical tests is shown in Figure 1.

**Extraction and purification of extracellular vesicles**

**Fig. 2:**
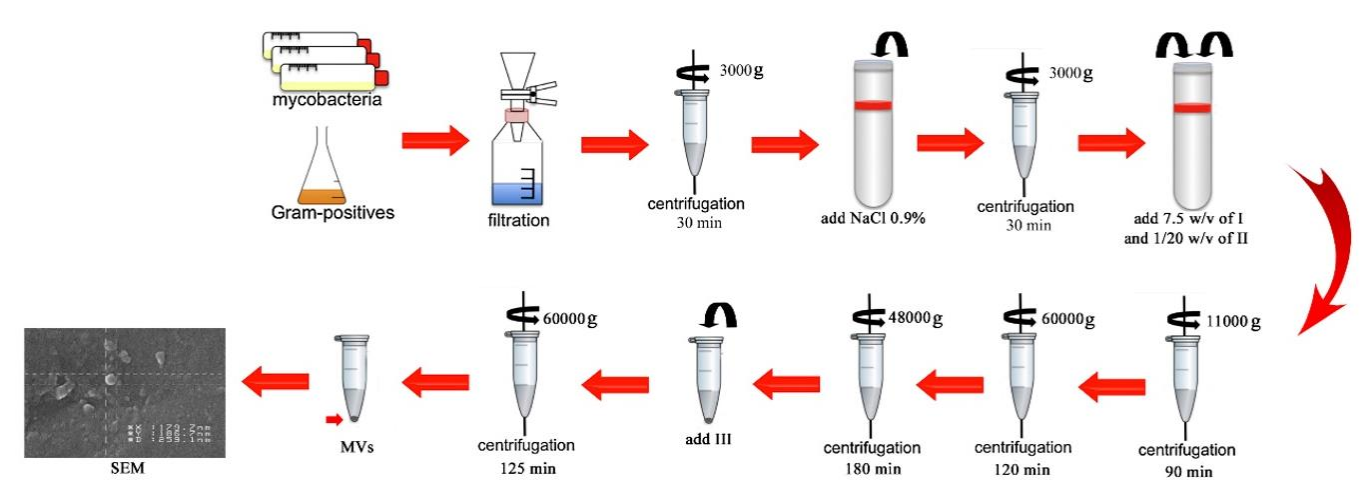
**Method of extraction and purification of** ***M. avium*** **extracellular vesicles.** The extraction solutions: I) 0.1 M buffer with 10 mM EDTA; II) 0.1 M Tris buffer with 10 mM EDTA and 100 g/l sodium deoxycholate; III) 0.1 M Tris buffer with 10 mM EDTA and 5 g/l sodium deoxycholate

Claassen is a method with some modifications was used to extract EVs (28). In summary, the culture medium of MAC was centrifuged at 3000×g for 30 min at 20^ o^C. Deactivation of MAC was performed at 80^ o^C, and the sediment was passed through a 0.22 µm polyvinylidene difluoride filter (Millipore, Billerica, MA). After that, 1 ml of 0.9% NaCl was added to the sediment. Then, the solution was centrifuged at 3000×g for 30 min at 4 ^o^C. The pellet was re-suspended in the solution I (0.1 M buffer solution with 10 mM EDTA) for 7.5 times of the pellet weight and blended slowly for 30 min. Next, a 1/20^th^ volume of solution II (0.1 M Tris buffer with 10 mM EDTA and 100 g/l sodium deoxycholate) was added to the mix, and blended extremely for 10 min. The suspension was centrifuged at 11000 × g for 90 min at 4^ o^C. The supernatant was centrifuged at 60000×g for 120 min at 4^ o^C, and then centrifuged again at 48000×g for 180 min at 4^ o^C. One ml of solution III (0.1 M Tris buffer with 10 mM EDTA and 5 g/l sodium deoxycholate) was added to 0.5 ml of the final solution, and was centrifuged at 60000×g for 125 min at 4 ^o^C. The pellet contained EVs. The concentrated EVs were re-suspended in 3% sucrose solution. The summary of EVs extraction procedure is shown in figure 2. Sample 1 was the EVs sediment and sample 2 was EVs supernatant at the last stage.

**Protein analysis of** **extracellular vesicles**

Samples were analyzed by 12% sodium dodecyl sulfate-polyacrylamide gel electrophoresis (SDS-PAGE) to evaluate the quality of total proteins. After electrophoresis by SDS-PAGE, proteins were stained with 0.1% (w/v) Coomassie Brilliant Blue. Also, Bradford assay (Bio-Rad, Hercules, CA, USA) and NanoDrop Lite spectrophotometer (Thermo Scientific, Wilmington, DE, USA) were used to measure the amount of proteins according to manufacturer’s instruction.

**Scanning electron microscopy**

To investigate the purity and stability of the natural form of the EVs at different stages of the purification process, scanning electron microscopy (SEM) was used. Five hundred µl of EVs was fixed on a foil on the slide surface for the preparation of gold coating. The gold particle was coated on samples onto the SEM sample stage in a vacuum, then high energy electrons were shot into the sample. DSR1 model that is a desk sputter coater of Nano-structured Coatings Company was used for gold sputtering system, and HITACHI S-4160 field emission scanning electron microscope was used for SEM.

**Endotoxin determination**

The Limulus Amebocyte Lysate (LAL) assay was used to measure endotoxin levels. Pierce® LAL chromogenic endotoxin quantitation kit (Thermo Scientific, USA) was used for endotoxin test. First, the microplate was kept for 10 min at 37 ºC. After that, 50 μl sample was added into the microplate well, the plate was covered with the lid and incubated for 5 min at 37 ºC. Fifty μl of LAL was added to each well and the plate was covered and gently shaken on a plate shaker for 10 s. Subsequently, the plate was incubated at 37 ºC for 10 min. One hundred μl per well of substrate solution was added, and the plate was covered with a lid and gently shaken. The plate was incubated at 37 ºC for 6 min. Fifty μl per well of stop reagent (or 25% acetic acid) was added to stop the color development, and the plate was shaken for 10 s. Finally, the absorbance was measured at 405-410 nm on a plate reader.

***In vivo*** **experiments**

To evaluate the effect of EVs on immune cells EVs were injected into 15 mice, six to eight weeks old, female, wild-type (BALB/c), and weighting 20 to 25 g that were purchased from Pasteur Institute of Iran. All mice were maintained under a specific-pathogen-free and biosafety level 2 conditions (29). One hundred and fifty µl of samples (50 µg/ml EVs) were injected (subcutaneous route) on day 0 and 14. Blood samples were collected on day 0, 14, and 28 from the sinuses located behind the orbit. On day 28, mice were sacrificed (cervical dislocation) and spleens were collected under sterile conditions. For anesthesia, 200 µl ketamine (130 mg/Kg) and xylazine (8.8 mg/Kg) diluted in phosphate-buffered saline (PBS) were injected (intraperitoneal route). This study followed all related national and international guidelines for animal studies and the Pasteur Institute of Iran IACUC approved the study.

**Quantification of total cytokines**

The spleen was placed in 5 ml RPMI 1640 (Pasteur Institute of Iran, Iran) media and mashed. This suspension was centrifuged at 1300 × g for 7 min at 4 ^o^C. Five ml of RBC lysis buffer was added to the sediment and centrifugation was repeated at the same speed. One ml RPMI 1640 media was added to the sediment. Ten µl of this solution was mixed with 10 µl trypan blue for cell counting. For cell challenge study, three concentrations of the antigens (EVs) (5, 10 and 20 µg/ml) were added to immune cells collected from mice spleen (2×10^6^ cells/ml). Cell culture was incubated at 37 °C with 5% CO_2_ for 72 h.

**ELISA tests**

Sandwich ELISA was performed to detect and measure IL-10 and IFN-γ according to manufacturer instructions with standards and samples in duplicate (Mabtech, Sweden). First, for the IFN-γ assay, the strips were washed 5 times with 300 μl dilution wash buffer per well. One hundred μl diluted standard was added to each well, and for the samples 50 μl of the samples with 50 μl buffer were added to each well. Following that, 100 μl biotinylated anti-mouse IFN-γ monoclonal antibody was added to each well, and the plate was covered and incubated at room temperature for 60 min. After washing, 100 μl streptavidin-horseradish peroxidase conjugate was added to each well, and the plate was covered. One hundred μl tetramethyl benzidine substrate was added to each well, and incubated at room temperature for 15 min in a dark place. One hundred μl stop solution (or 25% acetic acid) was added to each well to stop color development. Finally, the absorbance was measured at 450 nm. All of the above steps were repeated for the IL-10 assay.

**Statistical analysis**

Non-parametric tests were performed to compare the cytokine levels by SPSS software version 14.0. For all comparisons, a P-value <0.05 was considered as statistically significant.

## Results

**Extracellular vesicles characterization and identity test**

**Fig. 3:**
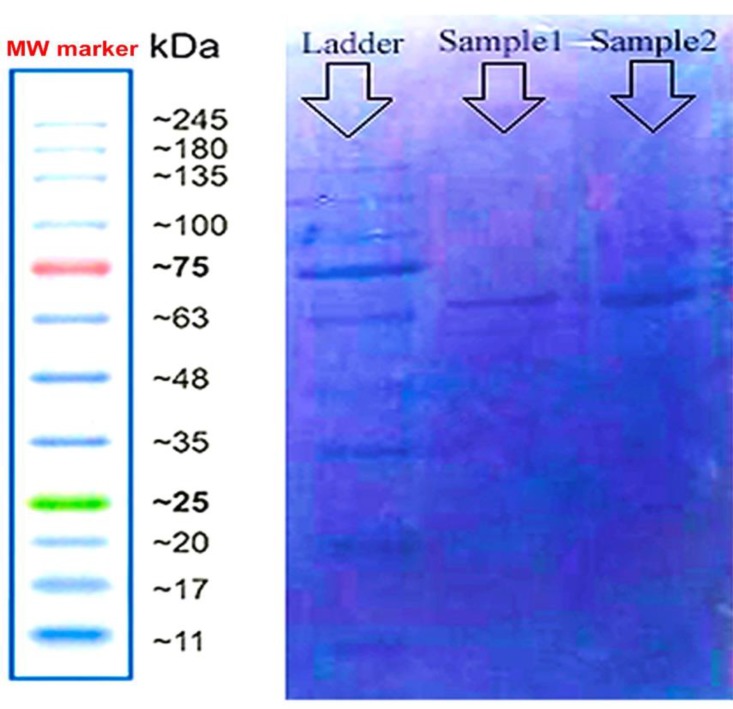
**Protein electrophoretic mobility view in 12% SDS-PAGE.** Sample 1: extracellular vesicles sediment; sample 2: extracellular vesicle supernatant; MW: molecular-weight size marker (CinnaGen, Cat. No. PR901641-tris-glycine 4-20%). For both samples, 62-70 kDa bands are visible

Twelve percent SDS-PAGE revealed 62 to 70 kDa bands as expected, although not very sharp. Sample 1 corresponded to pure EVs of MAC, and sample 2 was the supernatant of MAC (Figure 3). The bands pattern of both samples was similar. The 62-75 kDa region in the SDS-PAGE was identified as extracellular vesicle surface proteins.

The concentration of the proteins in the extracted EVs was 50 µg/ml.

**Scanning electron microscopy**

**Fig. 4:**
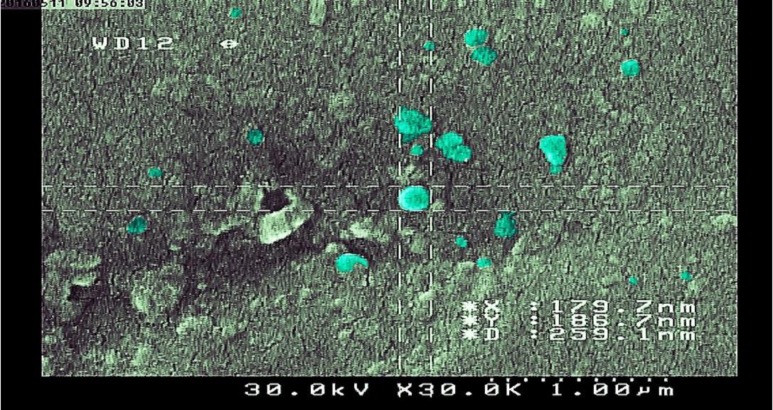
Scanning electron microscopy of extracellular vesicles. The diameter of EVs is between 73 to 295 nm. The vesicles were similar and stained randomly with the computer

SEM analysis of pellets obtained from MAC culture supernatants showed that the EVs were 73 to 295 nm in diameter (Figure 4). EVs kept their spatial characteristics after extraction and purification, and were completely spherical.

**Biological activity of the endotoxin**

The final pool of samples of EVs in a fivefold dilution contained 0.86 EU/ml. Therefore, the LAL values were less than 1. Zero EU/ml was defined as negative. 

**Cytokine assays**

*Mycobacterium avium *complex EVs induced a higher IL-10 than IFN-γ in the spleen immune cell culture. On average, the concentration of IL-10 was 300 µg/ml whereas IFN-γ was not detected (P = 0.037). Also, the concentration of negative control

(PBS) was 10 µg/ml for IL-10 and IFN-γ.

**Fig. 5:**
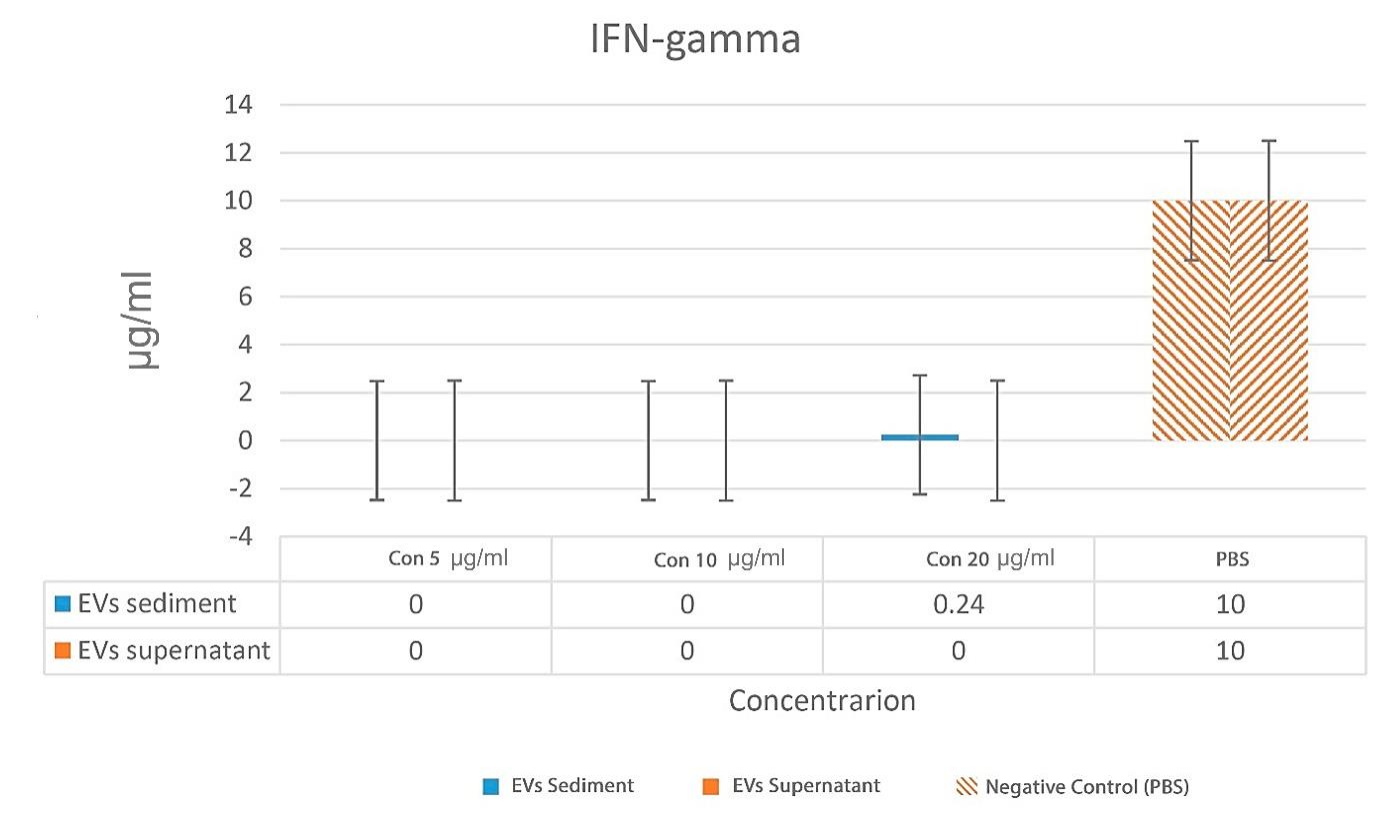
**Comparison of IFN-γ concentration for two samples with different EVs concentrations. **EVs: extracellular vesicles; con: concentration..The results were compared with PBS (*: P < 0.05)

**Fig. 6:**
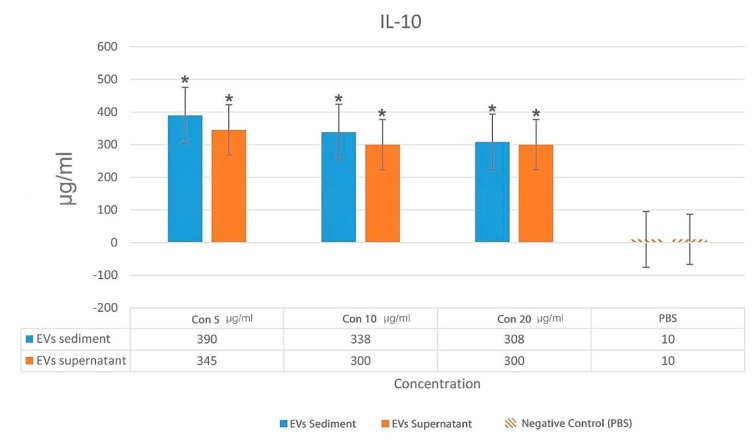
Comparison of the IL-10 concentration for two samples with different EVs concentrations. EVs: extracellular vesicles; con: concentration. The results were compared with PBS (*: P < 0.05)

IFN-γ concentrations for 5, 10, and 20 µg/ml of EVs are illustrated in Figure 5. The results demonstrate that the EVs did not trigger IFN-γ production even with high EVs concentration. IL-10 concentrations for 5, 10, and 20 µg/ml of EVs are illustrated in Figure 6. There was a statistically significant relationship between the levels of IL-10 with PBS (P =0.034).

## Discussion

Claassen’s method is an acceptable method to extract EVs from mycobacteria. It is technically easy, efficient, and cost-effective. The concentration of the proteins in the Bradford and NanoDrop tests was 50 µg/ml, confirming the accuracy and precision of the extraction method. Spatial characterization of EVs by SEM showed that their diameter was between 73 to 295 nm, similar to the previous studies (30).

Siadat et al. have recently used the same method to extract EVs from *Mycobacterium kansasii* with more than 5 prominent protein bands and a diameter of 200 to 300 nm (30).

In a study by Prados-Rosales et al. EVs generated from *M. tuberculosis* H37Rv strain led to the production of IFN-γ and IL-2 by spleen cells. They showed a high level of IFN-γ response to EVs of *M. tuberculosis* that was twofold higher than EVs of bacillus Calmette-Guerin (BCG). The SDS-PAGE results reported 20, 25 and 50 kDa bands and 90% of EVs were intact in the electron microscopy (31). In the present study, we found that the protein band of MAC EVs had about 62-75 kDa weight and was different from* M. tuberculosis* H37Rv.

EVs may be considered as a potential vaccine (32). A study of the immunogenicity of the inactive vaccine for prevention of tuberculosis in patients suffering from AIDS concluded that 19 recipients of EV vaccine had a higher response than control vaccine recipients after 3 to 5 doses of the vaccine in a year (33).

The current study found that spleen immune cells produced a Th-2 response with a high concentration of IL-10 and low concentration of IFN-γ. Finding the reason for increasing IL-10 requires further investigation. There are paradoxical reports on EVs immunogenicity. Kim et al. reported that the EVs of *Staphylococcus aureus *could stimulate a Th-1 response (34).

IL-10 has a strong immunomodulatory role and is a suppressor of IL-12 in *S. aureus* (35). If the anti-inflammatory response of MAC EVs can be repeated in other studies, we might find a new therapeutic agent for inflammation.

Since EVs play various roles in pathogenicity and signaling of bacteria, the current study of MAC EVS may open new perspectives for further scientific advances in detection, prevention, and treatment of infections caused by EVs producing bacteria. Our study may also suggest that EVs can be used for vaccines.

Our study suffers from a relatively small sample size of mice, and limitation in the number of measured cytokines. Therefore, further study is required to investigate the effect of MAC EVs on a broader spectrum of intracellular cytokines and their receptors. It is also important to determine the effect of EVs on intracellular gene expression profiles of macrophages and T-cells.
